# Species distribution and susceptibility profile of *Candida *species in a Brazilian public tertiary hospital

**DOI:** 10.1186/1756-0500-3-1

**Published:** 2010-01-03

**Authors:** Ariane Bruder-Nascimento, Carlos Henrique Camargo, Maria Fátima Sugizaki, Terue Sadatsune, Augusto Cezar Montelli, Alessandro Lia Mondelli, Eduardo Bagagli

**Affiliations:** 1 Botucatu Biosciences Institute, Sao Paulo State University, Botucatu, Brazil; 2 Medical School, Sao Paulo State University, Botucatu, Brazil

## Abstract

**Background:**

Species identification and antifungal susceptibility tests were carried out on 212 *Candida *isolates obtained from bloodstream infections, urinary tract infections and dialysis-associated peritonitis, from cases attended at a Brazilian public tertiary hospital from January 1998 to January 2005.

**Findings:**

*Candida albicans *represented 33% of the isolates, *Candida parapsilosis *31.1%, *Candida tropicalis *17.9%,*Candida glabrata *11.8%, and others species 6.2%. In blood culture, *C. parapsilosis *was the most frequently encountered species (48%). The resistance levels to the antifungal azoles were relatively low for the several species, except for *C. tropicalis *and *C. glabrata*. Amphotericin B resistance was observed in 1 isolate of *C. parapsilosis*.

**Conclusions:**

The species distribution and antifungal susceptibility herein observed presented several epidemiological features common to other tertiary hospitals in Latin American countries. It also exhibited some peculiarity, such as a very high frequency of *C. parapsilosis *both in bloodstream infections and dialysis-associated peritonitis. *C. albicans *also occurred in an important number of case infections, in all evaluated clinical sources. *C. glabrata *presented a high proportion of resistant isolates. The data emphasize the necessity to carry out the correct species identification accompanied by the susceptibility tests in all tertiary hospitals.

## Findings

Infections caused by opportunistic pathogens, such as yeasts, are becoming important causes of morbidity and mortality in many patients, because of alterations in the immune system and invasive hospital procedures [1]. Candidemia is commonly associated with high morbidity and mortality resulting in significant increases in the length of patients' hospitalization and in healthcare costs [2].

In the past two decades, nosocomial yeast infections have increased significantly worldwide [3]. In the United States, yeast infection ranks as the 4th most common cause of nosocomial bloodstream infection (BSI) [3]. In Brazil, *Candida albicans, Candida tropicalis *and *Candida parapsilosis *are the most common species isolated from BSI in several medical centers [2,4,5]. There has been an important shift in the species causing nosocomial candidemia, with the emergence of non-*albicans *species, particularly those more resistant to antifungal drugs [6,7]. Although studies demonstrate that antifungal resistance is relatively rare [2,4,8], antifungal drugs have been used intensively either to control such infections or as prophylactic in long-term treatments, creating serious worries that might select for drug resistances, thus greatly harming infection control [9,10]. *Candida *species have various degrees of susceptibility to the frequently used antifungal drugs. For example, while *Candida krusei *is intrinsically resistant to fluconazole, *Candida glabrata *is less susceptible or has higher MICs than other *Candida *species [10], which makes the correct species identifications and susceptibility tests pressing necessities.

In the present work, we present data on species frequency and antifungal susceptibility of *Candida *isolates obtained in a Brazilian public tertiary hospital.

## Results

### Species identification

Table 1 demonstrates the species distribution of *Candida *isolates. In a total of 212 yeast cultures, 70 (33%) were isolates of *C. albicans*, 66 (31.8%) *C. parapsilosis*, 38 (17.9%) *C. tropicalis*, 25 (11.8%) *C. glabrata*, 10 (4.7%) *C. guilliermondii*, 2 (0.9%) *C. lusitaniae *and 1 (0.5%) *C. pelliculosa*. With regard to clinical materials, *C. parapsilosis *was the species most commonly isolated from bloodstream infections (BSI) and also from peritoneal fluid (PF), while *C. albicans *presented a homogeneous distribution among the three sources, BSI, PF and urinary tract infections (UTI). *C. tropicalis *and *C. glabrata *were observed mainly in UTI isolates.

**Table 1:** Distribution frequency of *Candida *species obtained from different clinical materials at the Brazilian Tertiary Hospital (Clinical Hospital of the UNESP School of Medicine, Botucatu, São Paulo State).

Species	BSI	UTI	PF	Total
	% (n)	% (n)	% (n)	% (n)
*C. albicans*	32.4 (33)	34.1 (29)	32.0 (8)	33.0 (70)
*C. glabrata*	4.9 (5)	23.5 (20)	-	11.8 (25)
*C. guilliermondii*	6.9 (7)	1.2 (1)	8.0 (2)	4.7 (10)
*C. lusitaniae*	2.0 (2)	-	-	0.9 (2)
*C. parapsilosis*	48.0 (49)	8.2 (7)	40.0 (10)	31.1 (66)
*C. pelliculosa*	1.0 (1)	-	-	0.5 (1)
*C. tropicalis*	4.9 (5)	32.9 (28)	20.0 (5)	17.9 (38)
Total	102	85	25	212

### Susceptibility tests

Susceptibility tests for fluconazole, itraconazole and amphotericin B were performed on 212 isolates of *Candida *species. Table 2 summarizes the MIC ranges that delimit inhibition of isolates at proportions of 50 and 90%, determined by visual inspection, after 48 h incubation. Among all evaluated isolates, including *C. glabrata*, 31(14.6%) were resistant to fluconazole, 43 (20.3%) to itraconazole and 1(0.5%) to amphotericin B. When excluding this species the resistant isolates decrease to 14 (7.8%) and 21 (11.2%) for fluconazole and itraconazole, respectively.

**Table 2:** *In vitro *activity of antifungal agents against *Candida *spp. isolates from different clinical materials at the Brazilian Tertiary Hospital (Clinical Hospital of the UNESP School of Medicine, Botucatu, São Paulo State), from 1998 to 2005.

Isolates (n)	Drugs^a^	Cumulative % of isolates susceptible at a MIC (g/ml) of^d^:													
		
		0.03^b^	0.06	0.125^c^	0.25^a^	0.5	1	2	4	8	16	>16^c^	32	64	>64^b^
*C. albicans *(70)	FLU			5.7	30.0	40.0	65.7	74.3	80.0	84.3	**92.9**				100
	ITR	57.1	71.4	84.3	87.1	88.6	88.6	**90.0**	90.0	90.0	90.0	100			
	AMB				21.0	64.0	100								
															
*C. glabrata *(25)	FLU							4.0			16.0		32.0	**92.0**	100
	ITR		4.0	4.0	8.0	12.0	32.0	48.0	56.0	60.0	76.0	**100**			
	AMB					16.0	**100**								
															
*C. guilliermondii *(10)	FLU							30.0	30.0	80.0	90.0	90.0	90.0	**100,0**	
	ITR	20.0	50.0	60.0	**90.0**	90.0	90.0	90.0	90.0	90.0	90.0	100			
	AMB				10.0	30.0	**100**								
															
*C. parapsilosis *(66)	FLU			1.5	3.0	10.6	33.3	69.7	89.4	**98.5**	98.5	98.5	98.5	100	
	ITR	69.7	**90.9**	97.0	97.0	98.5	98.5	98.5	98.5	98.5	98.5	100			
	AMB				1.5	6.1	**98.5**	100							
															
*C. tropicalis *(38)	FLU			2.6	7.9	31.6	44.7	60.5	76.3	81.6	81.7	81.8	81.9	81.10	**100**
	ITR	21.1	31.6	55.3	68.4	73.7	78.9	84.2	84.2	84.2	84.2	**100**			
	AMB					21.0	**100**								
															
All *Candida *species^e ^(212)	FLU^a^			2.8	11.3	17.9	37.7	57.1	67.9	80.7	86.3	88.2	**94.3**	100	
	ITR^b^	45.3	61.3	72.2	77.8	79.7	83.0	86.3	87.3	87.7	**90.1**	100			
	AMB				8.0	30.7	**99.5**	100							
															

^a ^FLU: fluconazole, ITR: itraconazole, AMB: amphotericin B; ^b ^Fluconazole drug concentrations was evaluated from 0.125 to 64 μg/ml; ^c ^Itraconazole and amphotericin B drug concentrations were evaluated from 0.03 to 16 μg/ml; ^d ^Values corresponding to MICs at which at least 50% of isolates are inhibited are listed in underlined type and 90% in bold type; ^e ^Included 2 *C. lusitaniae *(FLU 2.0, ITRA 0.06; AMB 1.0) and 1 *C. pelliculosa *(FLU 2.0, ITRA 0.25; AMB 0.5).

Fluconazole exhibited the greatest activity against *C. albicans *with resistance observed in 5 (7.1%) isolates. Seven (18.4%) *C. tropicalis*, 1(1.5%) *C. parapsilosis*, 17 (68%) *C. glabrata *and 1 (10%) *C. guilliermondii *isolates were resistant to fluconazole. Resistance to itraconazole was found in 8 (10%) *C. albicans*, 22 (88%) *C. glabrata*, 2 (3%) *C. parapsilosis*, 10 (21.1%) *C. tropicalis *and 1 (10%) *C. guilliermondii *isolates. One *C. parapsilosis *isolate was amphotericin B-resistant. Isolates of *C. lusitaniae *and *C. pelliculosa *were susceptible to amphotericin B and to the azoles.

The MIC for fluconazole, itraconazole and amphotericin B of the QC strains ranged, respectively, from 1-4 μg/mL, 0.12-0.5 μg/mL and 0.5-1 μg/mL, for *Candida parapsilosis *ATCC 22019, and from 16-128 μg/mL, 0.25-1 μg/mL and 1-4 μg/mL for *Candida krusei *ATCC 6258.

## Discussion

The epidemiology of *Candida *infections has been extensively studied in North America and Europe [11], where large surveillance programs exist. In Latin America, these data are limited [2], with some regional studies in a few medical centers [4,5]. Colombo et al. [2] carried out the largest multicenter study in eleven medical centers of nine Brazilian cities; however, our hospital was not included in their study, and the data shown herein presented some peculiar differences both in the species frequency and in the susceptibility profile. The Botucatu Clinical Hospital is a regional state medical center that characteristically attends to a high proportion of patients from small communities and rural areas, with low access to medical assistance and low income, who are mainly in critical condition or in need of some advanced medical procedures, such as dialysis or chemotherapy. Consistent with several previous studies [2,4,5], the frequency of non-*albicans *species herein observed was greater than *C. albicans*. *C. parapsilosis *was the species most often isolated from BSI and PF, whose frequencies (43 and 40%) were higher than those observed in the previous Brazilian multicenter studies (7-40% in BSI) [2,4,5]. A peculiar species distribution was found in relation to the clinical sources. While in BSI and PF *C. parapsilosis *appears as the leading species, followed by *C. albicans *and *C. tropicalis*, in UTI, *C. albicans *occurs more frequently, followed by *C.tropicalis *and *C. glabrata*. Our findings confirm other studies that indicate *C. parapsilosis *as one of the most important species causing candidemia [2-8]. At the same time, the data also indicate that *C. glabrata *occurs less frequently, in substantial contrast to temperate countries of North America and Europe [11]. The predominance of *C. parapsilosis *in the peritoneal fluid under our casuistry also comes as no surprise, considering that this species appears to be common mainly in Latin America, and in other countries in patients receiving peritoneal dialysis [12,13]. The reasons why *C. parapsilosis *occurs more frequently in Latin American countries is not completely understood. *C. parapsilosis *is considered a commensal of human skin since it has been isolated from the hands of health workers [14], who have been identified as the major vectors in the infection acquisition [15]. At the same time, other local epidemiological factors also may make important contributions to the high frequency of *C. parapsilosis *in BSI and PF, such as a high proportion of neonates in the casuistry, as suggested by Weems [16], as well as the intense use of vascular catheters, parenteral nutrition and peritoneal dialysis procedures [17].

The isolation of *C. pelliculosa*, the asexual form of *Pichia anomala*, and *C. lusitaniae*, both rarely causing BSI, was found in other medical reports from Brazil [2,18] and other countries [19,20]. *C. guilliermondii*, also considered a normal component of human skin and mucosal flora and less common in the northern hemisphere, has been more frequently isolated in Latin America and presented reduced susceptibility to fluconazole [21,11,2].

In the present study, most of the isolates were susceptible to the antifungal drugs tested. Resistance to fluconazole and itraconazole was observed relatively high, mainly in isolates of *C. glabrata, C. tropicalis *and *C. albicans*. Similar to other studies, the percentage of isolates resistant to fluconazole was smaller than to itraconazole [22,23]. As expected, high secondary resistance rates were observed in *C. glabrata *to fluconazole (68%) and itraconazole (88%); this resistance to multiple azoles has been explained by an upregulation of CDR genes that encode the CDR efflux pumps [24]. Herein one of nine isolates of *C. guilliermondii *presented resistance to the both azole drugs, and high levels of resistance in *C. guilliermondii *has been observed worldwide [25]. The fact that resistance to amphotericin B was observed in one isolate of *C. parapsilosis *is controversial since most studies report a lack of amphotericin B resistance in *Candida *species [2,20,26], while other studies also found resistance to this drug in *C. parapsilosis *[4,27]. Amphotericin B is used most commonly in several Brazilian public tertiary hospitals in the treatment of systemic mycosis, in which the patients remain hospitalized for long periods of treatments, as in our hospital for paracoccidiodomycosis [28]. The possible effect of this drug against selectively resistant *Candida *species should not be excluded and merits proper evaluation.

In conclusion, the species distribution and antifungal susceptibility observed herein present several epidemiological features common to those observed in other tertiary hospitals in various Latin American countries, although also exhibit some peculiarities, such as a very high frequency of *C. parapsilosis *both in BSI and PF. *C. albicans *continues to occur in an important number of infection cases, with homogeneous distribution among all the evaluated clinical sources. *C. glabrata *presents a high proportion of resistant isolates, which reinforces the necessity to carry out the correct species identification in association with the susceptibility tests.

## Methods

### Origin of isolates

A total of 212 clinical isolates of *Candida *spp., isolated from bloodstream infections - BSI (102 isolates), urinary tract infections - UTI (85 isolates) and and peritoneal fluid - PF (25 isolates), obtained from patients from Clinical Hospital of the UNESP School of Medicine, Botucatu, São Paulo State, between January 1998 and January 2005 were evaluated in the study. The criteria and/or condition for the selection of *Candida *isolates to be analyzed were: i) the patients must be presenting clinical evidence of infection; ii) the materials from blood and peritoneal fluid were always collected by sterile puncture; iii) the positive cultures both from blood and peritoneal fluid were obtained in BACTEC System (BD Microbiology, Cockeysville, MD), followed by plating culture and identification by microscopy, biochemical tests and VITEK·ONE^® ^(BioMérieux, Durham, NC); iv) for the urine, the patients also must present clinical evidence of infection, the materials were collected in sterile cups from midstream urine specimen obtained after cleansing the external urethral meatus, cultured in MacConkey (Oxoid, Basingstoke, UK), Lactose Electrolyte Deficient agar (CLED; Oxoid,. Basingstoke, UK) and Sabouraud dextrose (Oxoid, Basingstoke, UK) agar plates, with counts equal to or above 10^4 ^colonies per ml. The peritoneal fluid materials were collected from patients in continuous ambulatory peritoneal dialysis (CAPD) by sterile puncture and we did not include samples from drainage tubes or bags. Repetitive isolates from the same patient were not included. All isolates were stored, in vial tubes containing Brain Heart Infusion plus 10% glycerol, in a freezer at -80°C. At the moment of the study each isolate was cultured on Sabouraud dextrose agar plates at 35°C.

### Species identification

All *Candida *species isolates were re-identified based on colony morphology on Chromogenic agar (CHROMagar *Candida*, Difco), microscopy features on Corn-meal agar slide culture, as well as the assimilation and fermentation tests.

### Susceptibility testing

Reference antifungal susceptibility testing of all 212 isolates was performed by BMD (broth microdilution) exactly as described in CLSI document M27-A2 [29] against fluconazole (Pfizer, Sao Paulo, Brazil), itraconazole (Janssen, Beerse, Belgium) and amphotericin B (Sigma, St. Louis, MO, USA). The isolates were incubated at 35°C and the presence or absence of growth, after 48 h, was observed by visual inspection. The MIC endpoint for amphotericin B was considered the lowest tested drug concentration able to prevent any visible growth, while the MIC for azoles was considered the lowest tested drug concentration causing a significant reduction (approximately 50%) in growth compared to the growth of the drug-free positive control [29]. MIC interpretations follow the CLSI breakpoints [29] for fluconazol (≤8 ug/ml, susceptible; 16-32 ug/ml, SDD, ≥64, resistant) and itraconazole (≤0.125 μg/ml, susceptible; 0.25-0.5 ug/ml, SDD, ≥1, resistant). For amphotericin B, due to a lack of consensus about the definition of this drug's MIC, previous interpretative breakpoints described elsewhere [30] were employed (≤1 ug/ml, susceptible, ≥2, resistant).

### Quality control

QC was performed for BMD in accordance with CLSI documents M27-A2 [29] by using *Candida krusei *ATCC 6258 and *Candida parapsilosis *ATCC 22019.

## List of abbreviations

BSI: bloodstream infection; UTI: urinary tract infection; PF: peritoneal fluid; CAPD: continuous ambulatory peritoneal dialysis; BMD: Broth microdilution.

## Competing interests

The authors declare that they have no competing interests.

## Authors' contributions

ABN and CHC carried out the laboratory experiments, tabulated the data and drafted the manuscript. MFS, ALM and TS participated in the design of the study and in the discussion. ACM conceived the study. EB participated in its design and coordination and helped to draft the manuscript. All authors read and approved the final manuscript.
